# Hemiarthroplasty versus angle-stable locking compression plate osteosynthesis in the treatment of three- and four-part fractures of the proximal humerus in the elderly: design of a randomized controlled trial

**DOI:** 10.1186/1471-2474-13-16

**Published:** 2012-02-09

**Authors:** Paul A Verbeek, Inge van den Akker-Scheek, Klaus W Wendt, Ron L Diercks

**Affiliations:** 1 Department of Orthopedic Surgery University Medical Center Groningen P.O. Box 30.001 9700 RB Groningen, the Netherlands; 2 Department of Traumatology University Medical Center Groningen P.O. Box 30.001 9700 RB Groningen, the Netherlands

## Abstract

**Background:**

The optimal surgical management of dislocated three- and four-part fractures of the proximal humerus in elderly patients remains unclear. Most used techniques are hemiarthroplasty and angle-stable locking compression plate osteosynthesis. In the current literature there is no evidence available presenting superior results between hemiarthroplasty and angle-stable locking compression plate osteosynthesis in terms of speed of recovery, pain, patient satisfaction, functional outcome, quality of life or complications.

**Methods/Design:**

A randomized controlled multicenter trial will be conducted. Patients older than 60 years of age with a dislocated three- or four-part fracture of the proximal humerus as diagnosed by X-rays and CT-scans will be included. Exclusion criteria are a fracture older than 14 days, multiple comorbidity, multitrauma, a pathological fracture, previous surgery on the injured shoulder, severely deranged function caused by a previous disease, "head-split" proximal humerus fracture and unwillingness or inability to follow instructions. Participants will be randomized between surgical treatment with hemiarthroplasty and angle-stable locking compression plate osteosynthesis. Measurements will take place preoperatively and 3 months, 6 months, 9 months, 12 months and 24 months postoperatively. Primary outcome measure is speed of recovery of functional capacity of the affected upper limb using the Disabilities of Arm, Shoulder and Hand score (DASH). Secondary outcome measures are pain, patient satisfaction, shoulder function, quality of life, radiological evaluation and complications. Data will be analyzed on an intention-to-treat basis, using univariate and multivariate analyses.

**Discussion:**

Both hemiarthroplasty and angle-stable locking compression plate osteosynthesis are used in the current treatment of dislocated three-and four-part fractures of the proximal humerus. There is a lack of level-1 studies comparing these two most-used surgical treatment options. This randomized controlled multicenter trial has been designed to determine which surgical treatment option provides the fastest recovery of functional capacity of the affected upper limb, and will provide better outcomes in pain, satisfaction, shoulder function, quality of life, radiological evaluation and complications.

**Trial registration number:**

The trial is registered in the Netherlands Trial Registry (NTR2461)

## Background

Proximal humeral fractures account for approximately 5% of all fractures [1]. More than 70% of patients with these fractures are older than 60 years of age, 75% are women, and the fractures are often related to osteoporosis [2,3]. For this elderly population the goal of treatment of proximal humeral fractures is to maintain independence of daily living by achieving a painless shoulder with an adequate function.

The majority of proximal humeral fractures are non- or minimally displaced, and are well treated nonoperatively [4,5]. In displaced three-and four-part proximal humeral fractures surgical treatment is recommended [5,6]. However, the optimal surgical management remains controversial. A wide variety of treatment modalities, varying from minimal percutaneous osteosynthesis to prosthetic replacement of the humeral head, has been described. Besides a certain consensus regarding prosthetic treatment of "head-split" fractures [6,7], the surgical treatment of choice is based on preference toward and experience with one of the treatment options. Most comminuted proximal humeral fractures are currently being treated with placement of a hemi-shoulder prosthesis, or in case of treatment of the humeral head with an angle-stable locking compression plate.

Specially designed prostheses have been developed for treatment of dislocated three-and four-part proximal humeral fractures. Important is the refixation and healing of the tuberosities [8-10]. Several studies report good outcomes regarding function, level of patient satisfaction, pain and complication rate [10-13]. Factors associated with poor outcomes are: secondary prosthetic replacement after initial osteosynthesis, malunion, osteolysis of the tuberosities, and excessive retroversion of the prosthetic head or over-lengthening due to prosthetic placement [14-18].

In recent years the use of angle stable locking compression plates has been popularized. Biomechanical data suggest that these implants can resist physiological loads in osteoporotic bone and may provide an alternative to hemiarthroplasty [19-21]. Other theoretical advantages compared to conventional plating are a reduction in screw loosening, less dissection of soft tissue, less compromising of periosteal vascularization through minimal plate pressure, and an increased primary stability which enables early functional mobilization. The clinical utility of angle-stable locking compression plates for three- and four-part fractures of the proximal humerus remains unclear [22-24]. Substantial rates of complications have been reported. Factors associated with poor outcomes are: the degree of varus malreduction, length of the initial metaphyseal hinge attached to the articular fragment and its correlation with osteonecrosis of the humeral head and screw protrusion through the humeral head.

A recent review could not draw firm conclusions with regard to which interventions are the most appropriate to manage different types of proximal humeral fractures [25]. Moreover, there are no level-1 randomized studies that compare surgical treatment of displaced three- and four-part fractures of the proximal humerus using hemiarthroplasty with surgical treatment using an angle-stable locking compression plate. It is therefore unclear which treatment leads to a better functional result: surgical treatment performing hemiarthroplasty or humeral head treatment with an angle-stable locking compression plate.

We designed a multicenter randomized controlled trial to compare hemiarthroplasty with an angle-stable locking compression plate for the treatment of dislocated three- and four-part fractures of the proximal humerus in an older population. This paper reports the study design of the HOMERUS (**H**emiarthroplasty vs. **O**steosynthesis in Hu**ME**ral F**R**act**U**re**S**) study.

## Methods/Design

### Study design

The HOMERUS study is designed as a level-1 multicenter prospective randomized controlled trial. Four centers in the Netherlands will participate. Eligible patients attending the Emergency Room (ER) with a comminuted proximal humeral fracture will receive information about the trial at the ER. After written consent has been confirmed, eligible patients will be randomly allocated to the two different types of surgical treatment. To keep the allocation of patients in different clinics even, block randomization will be used. For every participating hospital an exclusive sequence will be used and every block will contain ten patients. Randomization will be accomplished via opaque, sealed envelopes. Follow-up will take place over a period of two years. The inclusion period is planned from January 2011 to December 2012. The study design, procedures, protocols and informed consent are approved by the Medical Ethical Committees of the participating hospitals. The trial is registered in the Netherlands Trial Registry (NTR2461), and is designed in accordance with the Declaration of Helsinki [26] and the Medical Research Involving Human Subjects Act. It will follow the CONSORT (Consolidation of Standards of Reporting Trials) guidelines [27,28].

### Study population

All patients attending the ER with a three- or four-fragment fracture of the proximal humerus with dislocation on X-ray and who are older than 60 years of age will undergo further CT-scan examination. If the CT-scan shows more than 5 mm dislocation in one of the fracture planes [29], the patient is eligible for inclusion [table 1]. Patients with a fracture more than 14 days old, multiple comorbidity, multitrauma (Injury Severity Score > 15), pathological fracture, previous surgery on the injured shoulder, severely deranged function caused by a previous disease, head-split proximal humerus fracture [6,7], or unwillingness or inability to follow rehabilitation instructions are excluded.

**Table 1:** Inclusion and exclusion criteria

Inclusion criteria
3- and 4-fragment fracture of the proximal humerus
> 5 mm dislocation in one of the fracture planes
> age 60 years

**Exclusion criteria**

Fracture older than 14 days
ASA IV-V
Multitrauma ISS > 15
Pathological fracture
Previous surgery on injured shoulder
Severely deranged function caused by a previous disease
"Head split" type fracture of proximal humerus
Unwillingness or inability to follow instructions

### Interventions

Patients will be randomized to either hemiarthroplasty or angle-stable locking compression plate osteosynthesis.

**Figure 1:**
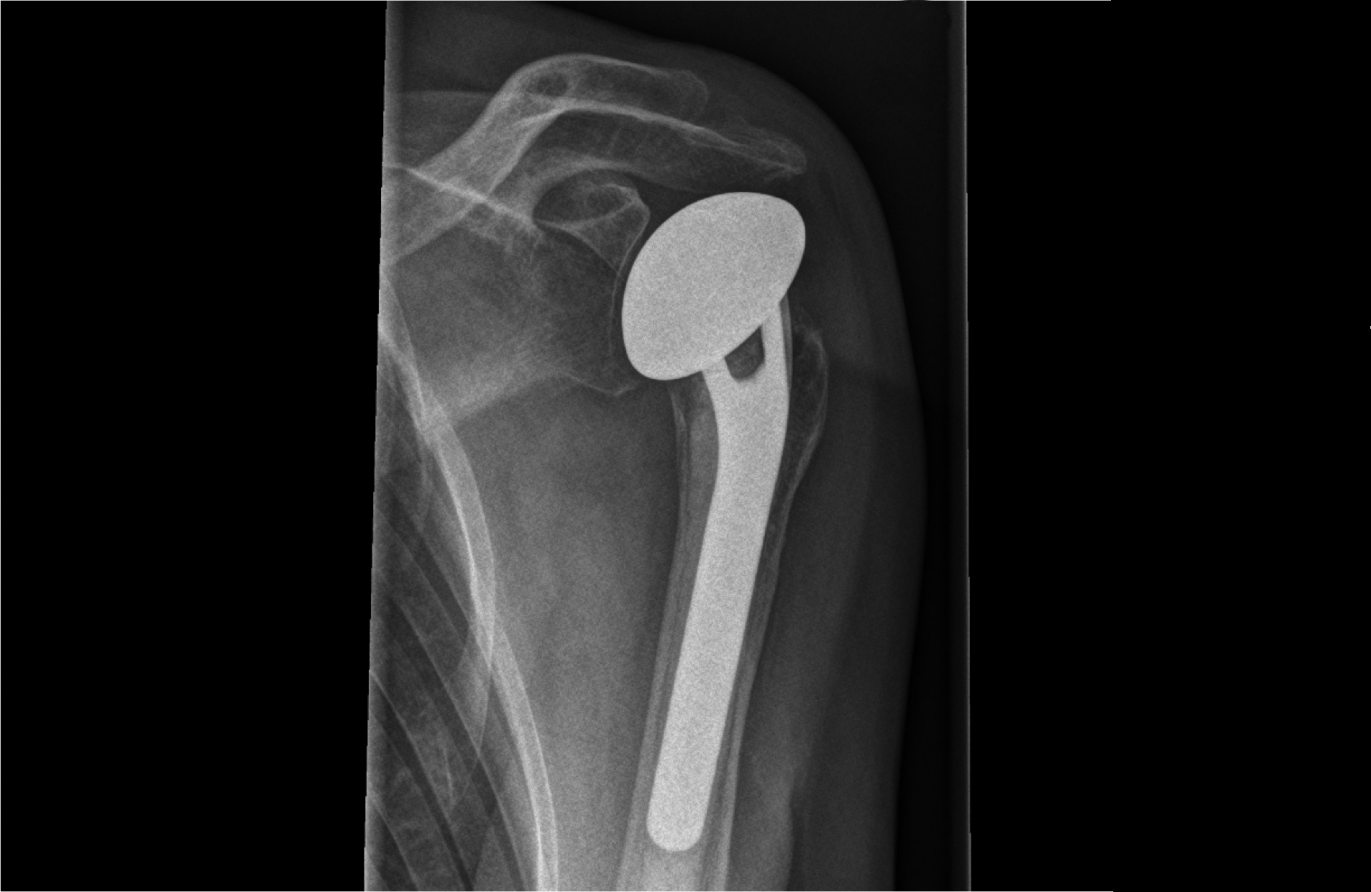
**Example of proximal humerus fracture, managed by hemiarthroplasty**.

Hemiarthroplasty [Figure 1] will be performed using the fracture shoulder prosthesis as provided at the participating hospital. A standardized deltopectoral approach with the patient in beach-chair position is used. After identification of the greater and lesser tuberosities, strong non-absorbable sutures are placed through the bone-tendon junctions. The head fragment is taken out and preserved for prosthetic head measurement. The humeral shaft is prepared for the specific implant. Humeral length is restored and the prosthesis is cemented in approximately 20-30 degrees retroversion. Tuberosity positioning is performed with the use of nonabsorbable sutures to attach the fragments to the prosthesis, the shaft, and to each other in an anatomic position.

**Figure 2:**
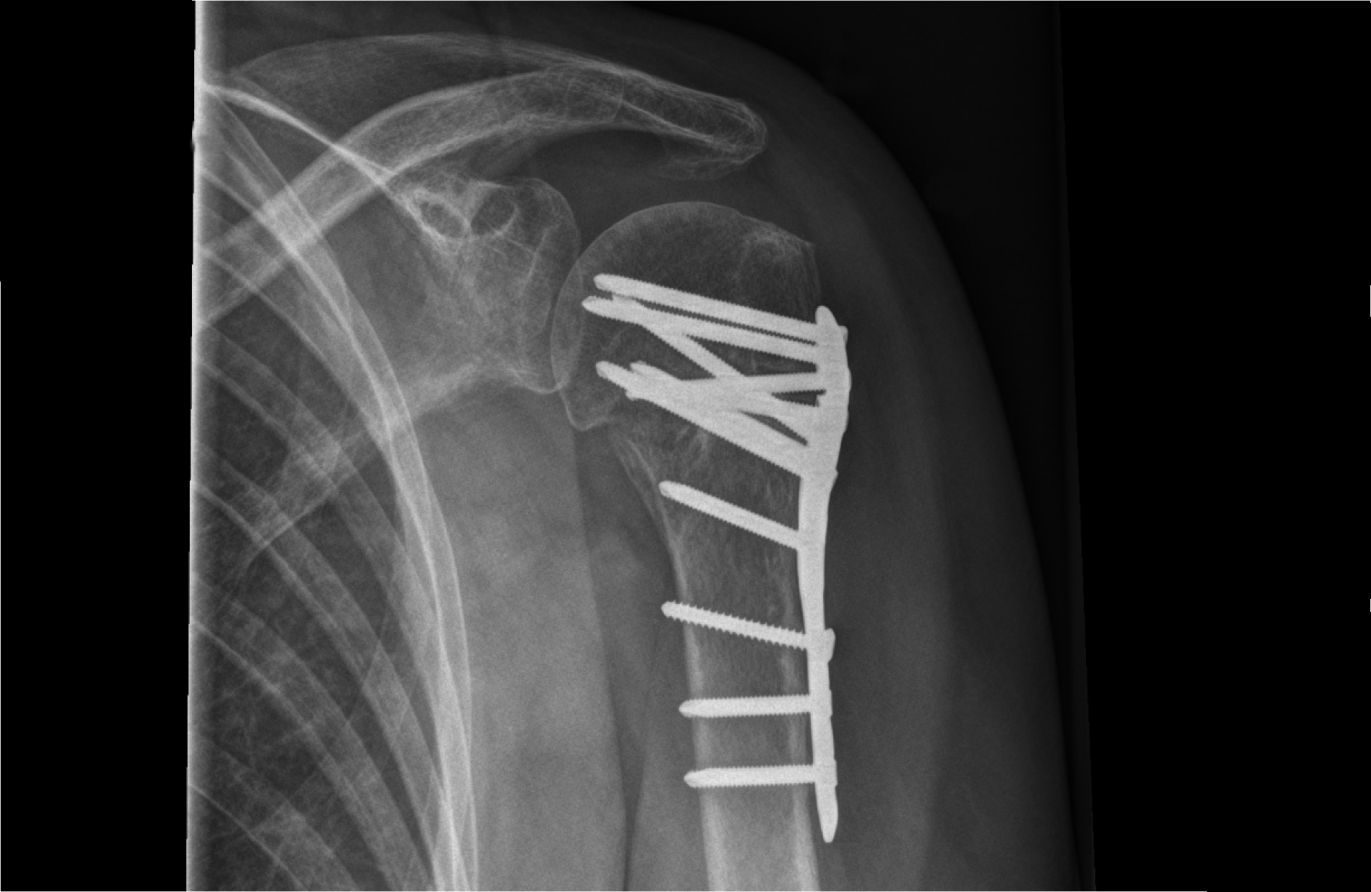
**Example of proximal humerus fracture, managed by angle-stable locking compression plate osteosynthesis**.

Angle-stable locking plate osteosynthesis [Figure 2] is performed with the patient in supine or beach-chair position on a radiolucent table and a deltopectoral approach is used. The fracture is reduced and provisionally stabilized with (threaded) Kirschner wires. The reduction is confirmed as adequate with use of image intensification. The angle-stable locking compression plate is positioned with the help of a mounted aiming device, at least 5-8 mm distally of the upper end of the greater tuberosity and 2 mm posteriorly to the bicipital groove. Care is taken to ensure that a sufficient gap is maintained between the plate and the tendon of the long head of the biceps. When fracture reduction and subsequent screw positioning is considered adequate, the plate is fixed definitively with the insertion of angular stable screws in the humeral head. The use of angular stable or standard cortical screws for the humeral shaft holes is left to the treating surgeon. A final image intensifier check to verify correct screw placement is performed.

The surgical procedures will be performed by a maximum of three qualified and experienced surgeons at each participating hospital. Surgery will take place within 2 weeks after the date of trauma. In both groups the specific type of implant will be determined by the attending surgeon at hospital the patient has been admitted to. In both groups a standard deltopectoral approach will be used. Antibiotic prophylaxis with first-generation cephalosporin will be given intravenously, preoperatively and for 24 hours postoperatively. Postoperatively patients will receive thromboprophylaxis during hospital stay (e.g. Low Molecular Weight heparin (LMWH) or equivalent). In terms of rehabilitation, all patients will be treated with a standardized protocol at each hospital. After surgery, the patient receives a sling for six weeks combined with mobilization instructions. Two weeks postoperatively, active range of motion will be increased to the horizontal level; after another two weeks active external rotation will be initiated. Rehabilitation will be supervised by a physiotherapist.

### Measurements

**Figure 3:**
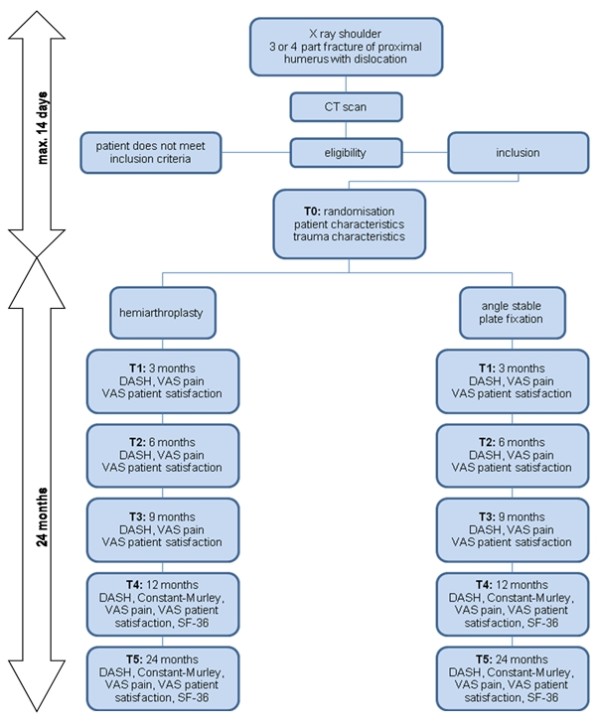
**Flowchart HOMERUS study**.

Outcome assessment will take place in both groups at randomization (T0), 3 months postoperatively (T1), and 6, 9, 12 and 24 months (T2, T3, T4, T5) postoperatively. Outcome assessment for T0, T1, T4 and T5 will take place in an outpatient clinic setting and involves all outcome measures. At six and nine months postoperatively (T2 and T3) patients will only fill in a questionnaire that will be sent and returned by mail [Figure 3]. Speed of recovery of functional capacity of the affected upper limb is our primary outcome measure. Secondary outcome measures are pain, patient satisfaction, shoulder function, quality of life, radiological evaluation and complications.

#### Functional capacity of the affected upper limb

The DASH score will be used to determine functional capacity [30,31]. This questionnaire consists of 30 questions about symptoms and function of the upper limbs that are affected by disorders of the musculoskeletal system. It provides a single main score, the DASH function/symptom score, which is a summation of the responses on a scale of 0 (no disability) to 100 (severe disability). The degree of difficulty in performing a variety of daily physical activities because of arm, shoulder or hand problems is determined (21 items), as well as the severity of pain, activity-related pain, tingling, weakness and stiffness (5 items), and the effect of the upper-limb problems on social activities, work, sleep and self-image (4 items).

#### Pain

The amount of experienced pain is measured on a 10-point Visual Analogue Scale (VAS), from no pain (0) to most extreme pain imaginable (10).

#### Patient satisfaction

The amount of experienced satisfaction with the result of surgical treatment is measured on a 10-point Visual Analogue Scale, from no satisfaction at all (0) to complete satisfaction (10).

#### Shoulder function

Functional outcome is measured with the Constant-Murley score. This score system combines shoulder function tests (65 points) with a subjective evaluation of the patients (35 points) [32].

#### Quality of life

Health-related quality of life is measured with the Short-Form 36 (SF-36) score. This is a validated multi-purpose, short-form health survey with 36 questions, representing eight health domains [33,34].

#### Radiological evaluation

Standard X-rays (40° posterior oblique view with external rotation of the humerus, 40° posterior oblique view with internal rotation of the humerus, and an axillary view) and computer tomography will be taken preoperatively and used for classification of fracture type and surgery-planning purposes. Postoperative imaging will consist solely of standard X-rays. Points of particular interest in hemiarthroplasty are signs of loosening; periarticular calcification; displacement, dislocation or necrosis of the tuberosities; glenohumeral subluxation; periprosthetic lucency; component shift; loss of glenoid cartilage; and presence of bony erosion on the glenoid. In angle-stable locking compression plate osteosynthesis axial and rotational deformities of the head fragment and/or greater tuberosity fragment after surgery will be assessed radiographically according to Bahrs et al. [35]. Secondary displacement of the fracture, screw perforation, humeral head necrosis classified according to Cruess [36], plate impingement and delayed fracture healing/pseudoarthrosis will also be recorded.

#### Complications

All complications will be recorded during the study period.

#### Sample size

Different studies on clinical outcomes following hemiarthroplasty in the treatment of three- and four-part fractures of the proximal humerus state that the DASH score is about 40 [37,38]. Sample size calculation was performed; primary outcome measure was the DASH score, whereby 10 points after 24 months was considered a clinically relevant difference between the two groups, with a standard deviation of 19.4, alpha set on 5% and power on 80%. This resulted in a required number of 61 patients in each group. Assuming a dropout rate of 10%, two groups of 67 patients will have to be included.

### Statistical analysis

To estimate the effect of the interventions, analyses will be performed using the Statistical Package for the Social sciences (SPSS) 19.0. The baseline characteristics from both study groups will be compared for equality by means of an independent samples T-test for continuous variables and a chi-square test for dichotomous variables. Random effect models will be applied for longitudinal analyses. A p-value lower than 0.05 will be considered as statistically significant. Data will be analyzed according to the intention-to-treat principle and the research data will be reported following the CONsolidated Standards of Reporting Trial (CONSORT).

## Discussion

Both hemiarthroplasty and angle-stable locking compression plate osteosynthesis for dislocated three-and four-part fractures of the proximal humerus in the elderly are performed in current practice. The optimal surgical management remains controversial and a clear distinction between indications for one of both treatment options cannot be made on the basis of the current evidence in the literature. To date, there are no randomized controlled studies that compare the outcome of hemiarthroplasty vs. angle-stable locking compression plate osteosynthesis in the treatment of dislocated three- and four-part fractures of the proximal humerus. The HOMERUS study is designed to determine which treatment results in better outcome, defined as speed of recovery of functional outcome. Further, it is possible to identify which treatment will provide better outcomes in pain, satisfaction, shoulder function, quality of life, radiological evaluation and complications.

## Competing interests

The authors declare that they have no competing interests.

## Authors' contributions

PAV and RLD originated the idea for the study, contributed to its design with KWW and IvA, and developed the intervention protocol. PAV is responsible for the data collection and drafted the manuscript. All authors have read, edited and approved the final manuscript.

## Pre-publication history

The pre-publication history for this paper can be accessed here:

http://www.biomedcentral.com/1471-2474/13/16/prepub
